# Heterogeneously catalyzed lignin depolymerization

**DOI:** 10.1007/s13203-016-0157-y

**Published:** 2016-06-03

**Authors:** Antonio Pineda, Adam F. Lee

**Affiliations:** European Bioenergy Research Institute, Aston University, Birmingham, B4 7ET UK

**Keywords:** Biomass, Lignin, Heterogeneous catalysis, Solid acid

## Abstract

Biomass offers a unique resource for the sustainable production of bio-derived chemical and fuels as drop-in replacements for the current fossil fuel products. Lignin represents a major component of lignocellulosic biomass, but is particularly recalcitrant for valorization by existing chemical technologies due to its complex cross-linking polymeric network. Here, we highlight a range of catalytic approaches to lignin depolymerisation for the production of aromatic bio-oil and monomeric oxygenates.

## Introduction

The concept of sustainability is now embedded throughout modern society, from the design of cities and methods of food production and transport, to the sourcing and recycling of natural resources. Integral to these developments are new technologies able to deliver chemicals and fuels from renewable, i.e., non-fossil, resources, of which biomass has emerged as one of the most abundant and economically attractive feedstocks. Lignocellulosic biomass, in particular, is considered an attractive source of biomass for low carbon energy thermal conversion technologies, such as pyrolysis and gasification, since its use, in this regard, faces only limited competition from the agricultural and paper sector. However, biomass as a replacement for fossil fuel feedstocks is problematic due to the high water and oxygen content of the former, the presence of a water-soluble fraction of alkaline and halogen elements, along with hazardous trace elements, and resultant low-energy density/heating value, pH, and ash-fusion temperatures of biomass. The heterogeneous nature of biomass between different plants and microorganisms, and significant regional and seasonal variations even within the same species, further necessitate the development of versatile processes for the chemical transformation of biomass [1]. However, there are strong political and financial drivers for renewable energy technologies, with the European Union mandating that a 20 % of overall energy consumption must derive from renewable sources by 2020, rising to 27 % by 2030. In regard of transportation fuels, 10 % must originate from renewable resources, and provide reductions in greenhouse gas emissions of at least 35 % as compared with fossil fuels (including emissions arising from cultivation, processing, and transport). Policy directives are also being introduced to ensure that land designated for biomass production for bio-fuels cannot have been previously used for carbon stock, such as wetlands or forest, or impact of regions with high biodiversity, such as primary forests or highly biodiverse grasslands [2].

**Fig. 1:**
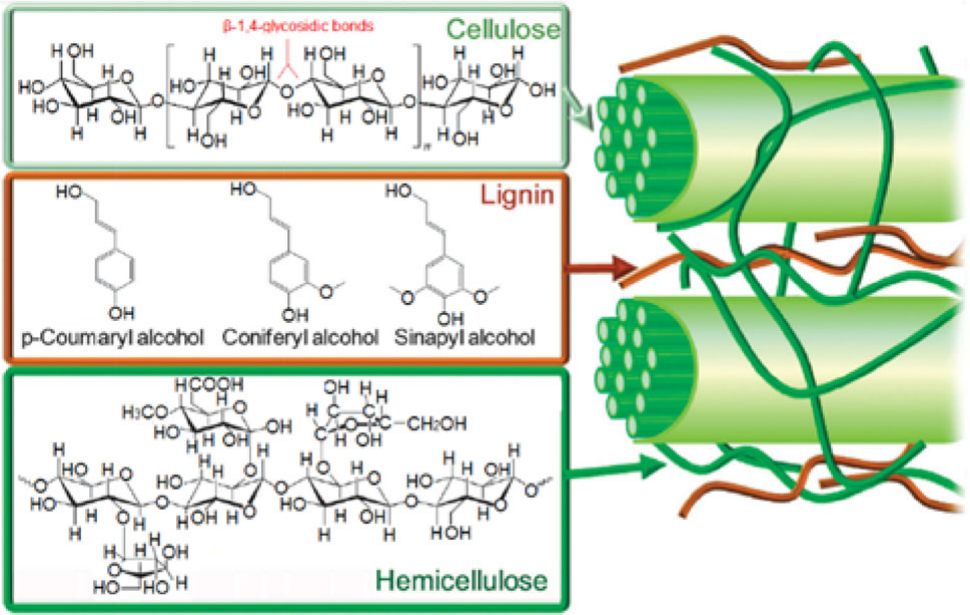
Schematic of cellulose, hemicellulose, and lignin components of lignocellulosic biomass and associated molecular building blocks. Reproduced from Ref. [8] with permission of The Royal Society of Chemistry 2012

Lignocellulosic biomass comprises lignin, cellulose, and hemicellulose, with lignin the second most abundant constituent after cellulose at around 10–30 wt% (Fig. 1). Lignin is a highly branched phenolic polymer of high molecular weight, typically between 600 and 1500 kDa [3], with an annual global production of around 40–50 million tons. It occurs primarily in plant cell walls, and the principal components are guaiacyl alcohol, syringyl alcohol, and p-coumaryl alcohol [4]. Lignin depolymerization can afford phenolic ‘platform chemicals’, and, consequently, has attracted a considerable attention for the production of both chemicals and second generation bio-fuels (as a non-edible biomass feedstock). The selective cleavage of C–O bonds that connect the monomers in lignin is a challenging, but offers compounds suitable for subsequent upgrading for fine chemicals applications, and has been achieved through solvolysis, hydrolysis, hydrogenolysis, pyrolysis, and alkaline oxidation. Oxidizing routes are generally undesirable due to radical formation which can result in partial repolymerization, whereas hydrogenolysis protocols minimize lignin repolymerization and promote C–O bond cleavage by quenching and recombination of radicals, although the addition of molecular hydrogen can result in fully hydrogenated cyclic hydrocarbons of relatively low commercial value. Hydrogen-donating solvents able to generate/transfer hydrogen have, thus, also shown promise, e.g., Connors et al. report hydrogen transfer from ethanol, tetralin, and formic acid during lignin hydrocracking [5], while Kleinert et al. reported a novel solvolysis method employing high-pressure thermal treatment of lignin in the presence of ethanol as solvent and formic acid as hydrogen-donor, the latter decomposing to liberate CO_2_ and hydrogen [3]. Glycerol [6] and isopropanol [7] are also promising hydrogen-donor solvents. Due to the wide range of possible products, and strength of the C–O bonds underpinning the lignin network, catalysts are essential to facilitate energy and atom-efficient lignin depolymerization, and selectively deoxygenate the resulting products for fuel applications.

Lignin valorization is the key to fuels and chemicals co-production from biomass through the biorefinery concept. Potential process under consideration within biorefineries fall into three broad classes, as categorized by Gallezot [9]: high-temperature thermal conversion via gasification or pyrolysis to deliver light gaseous or condensable molecules for subsequent transformation via established chemical processing, such as Fischer–Tropsch conversion of syngas; lower temperature catalytic or enzymatic conversion of lignin into aromatic building blocks, such as benzene, toluene, xylene, and phenol; or one-pot routes direct to highly functionalised products.

## The structure of lignin

**Fig. 2:**
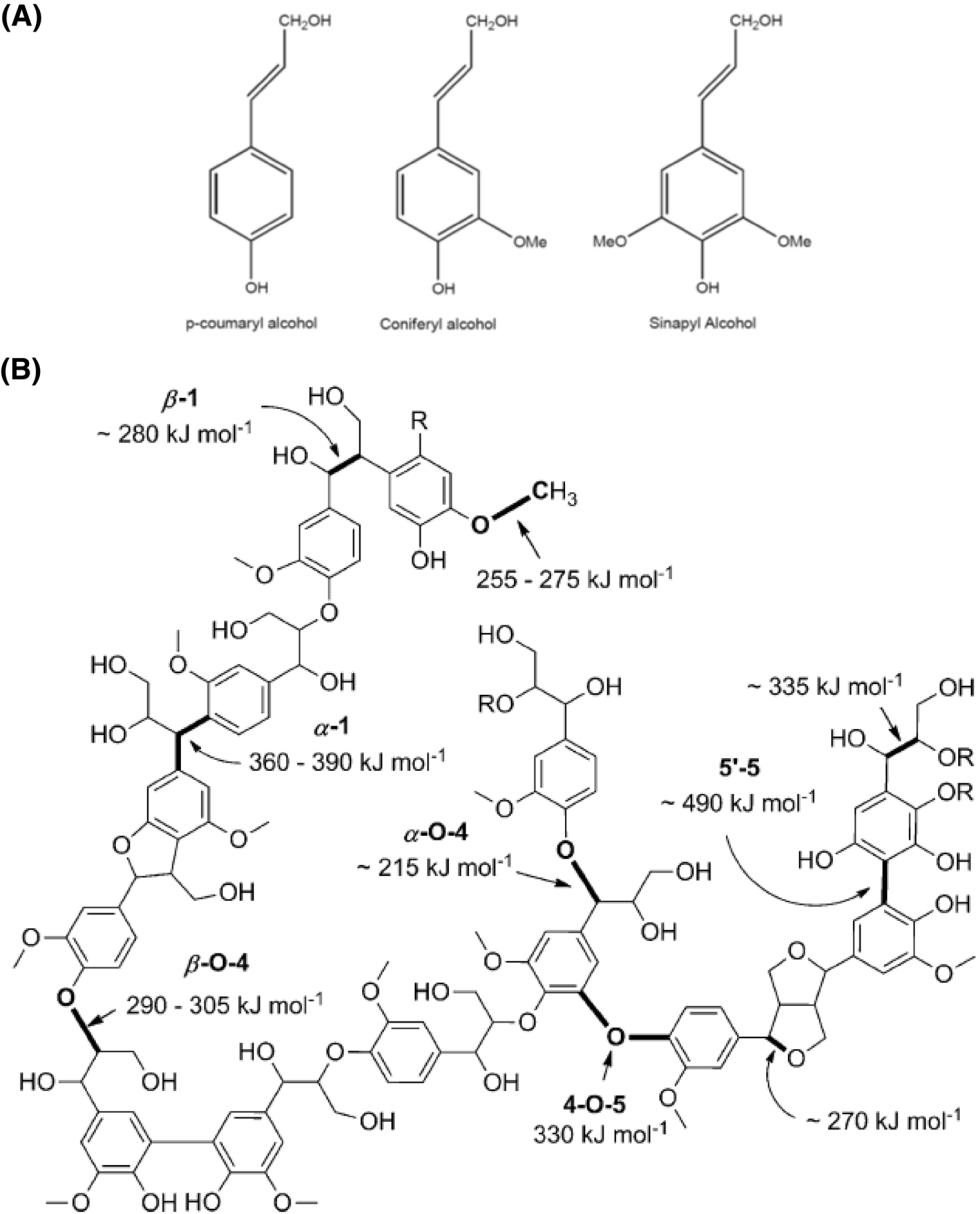
**a** Common building blocks of lignin and **b** exemplar polymer fragment of lignin and estimated bond strengths of ether linkers. Reproduced from Ref. [14] with permission of John Wiley & Sons 2012

Lignin is a three-dimensional amorphous polymer whose most important building blocks are guaiacyl alcohol, syringyl alcohol, and p-coumaryl alcohol (Fig. 2a), linked predominantly by β-O-4 bonds, in addition to 5-5, α-O-4, β-5, 4-O-5, β-1, dibenzodioxocin, and β-β bonds. The structure and, hence, physicochemical properties of lignin vary with the biomass source, and hence the relative abundance of these monomeric sub-units. Hence, softwood lignin typically comprises 90 % coniferyl alcohol, whereas similar amounts of coniferyl and sinapyl alcohol occur in hardwood lignins, with grass lignin a mix of coniferyl, sinapyl, and coumaryl alcohols [10, 11]. Figure 2b shows a lignin fragment and the estimated bond strengths of each ether linkage. The abundance of each bond type also varies with the lignin source, with around 39–48 % of linkers β-O-4 bonds in softwood, but somewhat lower for hardwoods (32–37 %) [12, 13]. The heterogeneity of phenolic constituents and linkages is a significant barrier in the design lignocellulosic of biorefineries, since it prohibits a single protocol for lignin processing.

## Delignification of biomass

Lignin isolation from lignocellulosic biomass is non-trivial due to competing condensation and oxidation reactions. Lignin is obtained commonly through either sulfur-derived technologies, such as those employing sulfites for Kraft lignins, or sulfur-free processes, in which lignin is extracted by organic solvents or alkaline solution. Additional methods employed to isolate lignin are indicated by the following nomenclatures: milled wood lignin, acidolysis lignin, cellulolytic enzyme lignin, enzymatic mild acidolysis lignin, pyrolysis lignin, and steam explosion lignin [15]. Alkaline lignin or Kraft lignin is produced in large quantities by the pulp and paper industry, which are, hence, the most abundant sources of lignin for the subsequent chemical processing [3]. In the Kraft process, lignocellulose is treated with either sodium hydroxide and/or sodium sulfide at 170 °C, facilitating the separation of cellulosic fibers from lignin [16]; however, this approach is energy intensive, and the sulphite route introduces considerable sulfur into the resulting lignin. The Organosolv process for lignin isolation was developed by Theodor Kleinert [17], and as its name suggests employs organic solvents to extract lignin, which consequently has a low ash and sulfur content compared with Kraft lignin. Depending on the severity of the treatment, partial dissolution of cellulose and hemicellulose may also occur [18]. The lignin extraction efficiency in an organic solvent, such as ethanol, can be increased by the addition of an inorganic acid, or by replacing ethanol with lower boiling point acetone which facilitates lignin recovery through simple distillation [18].

## Catalytic lignin depolymerization

The decomposition of lignin into its constituent phenolic components is a hindered by the complex interconnectivity within the polymeric structure [19]. Diverse methods have been employed to produce phenolic compounds from lignin, including hydrolysis, hydrocracking (hydrogenolysis), and oxidation [11, 20]. Oxidative cleavage of C–C and C–O–C bonds can afford vanillin and similar compounds accompanied by CO, CO_2_, and H_2_O formation. However, oxidative protocols are undesirable, because the resulting free radicals drive recondensation. In contrast, reductive cleavage of such bonds has proven more successful for yielding monomeric compounds, such as phenols, benzene, toluene, and/or xylene.

Homogeneous catalysts have been rarely utilised due to their inherent disadvantages in respect of their difficult separation from the reaction mixture, corrosiveness (in the case of soluble acid catalysts), and, hence, are more favoured for selective bond cleavage within model compounds, but have been implemented in oxidative depolymerisation [21, 22]. The present review focuses on reductive catalytic solutions which favour phenolic compounds suitable for subsequent upgrading by HDO routes [23] to yield fuels and fuel additives.

### Hydrogenolysis

Catalytic depolymerization is dominated by thermochemical transformations to monomeric phenols and typically requires harsh conditions due to the stability of the polyphenolic lignin structure. Organosolv lignin is more sensitive to hydrogenolysis due to its high degree of unsaturation and low molecular weight [24]. Bifunctional catalysts, containing both acid and noble metal sites, have attracted interest in this arena, since they can simultaneously cleave ether bonds and deoxygenate the resulting phenolic monomers in the presence of molecular H_2_ or a H-donor solvent. The use of noble metal catalysts favours a mixture of cyclohexane derivatives and products arising from C–C bond cleavage. Metallic nickel has shown the most promise for the production of phenolics from lignin due to its superior propensity for cleaving aryl–aryl C–O–C bonds and C–OH bonds in side chains containing CH_3_ or CH_2_ functions, commonly yielding C_1_–C_3_ alkane-substituted guaiacols as the major product [25]. In the case of lignosulfates, Ni catalysts show good resistance to sulfur poisoning, while remaining active and selective for the transformation of lignosulfate into phenolics. While nickel is able to cleavage C–O aliphatic bonds, it is less active for the rupture of aromatic C–O and arene bonds. Lignin conversion to alkane-substituted guaiacols over Ni catalysts is independent of the support type (e.g., activated carbon, zeolite, or MgO) [25]. Higher conversions were obtained in the presence of diol or triol functions, such us glycerol and ethylene glycol. Song and co-workers concluded that for lignosulfates, Ni(0) active sites catalyse hydrogenolysis of C–O–C bonds, C–OH bonds in side alkyl chains, and the reduction of sulfonate groups into hydrogen sulfide (H_2_S) [25].

**Fig. 3:**
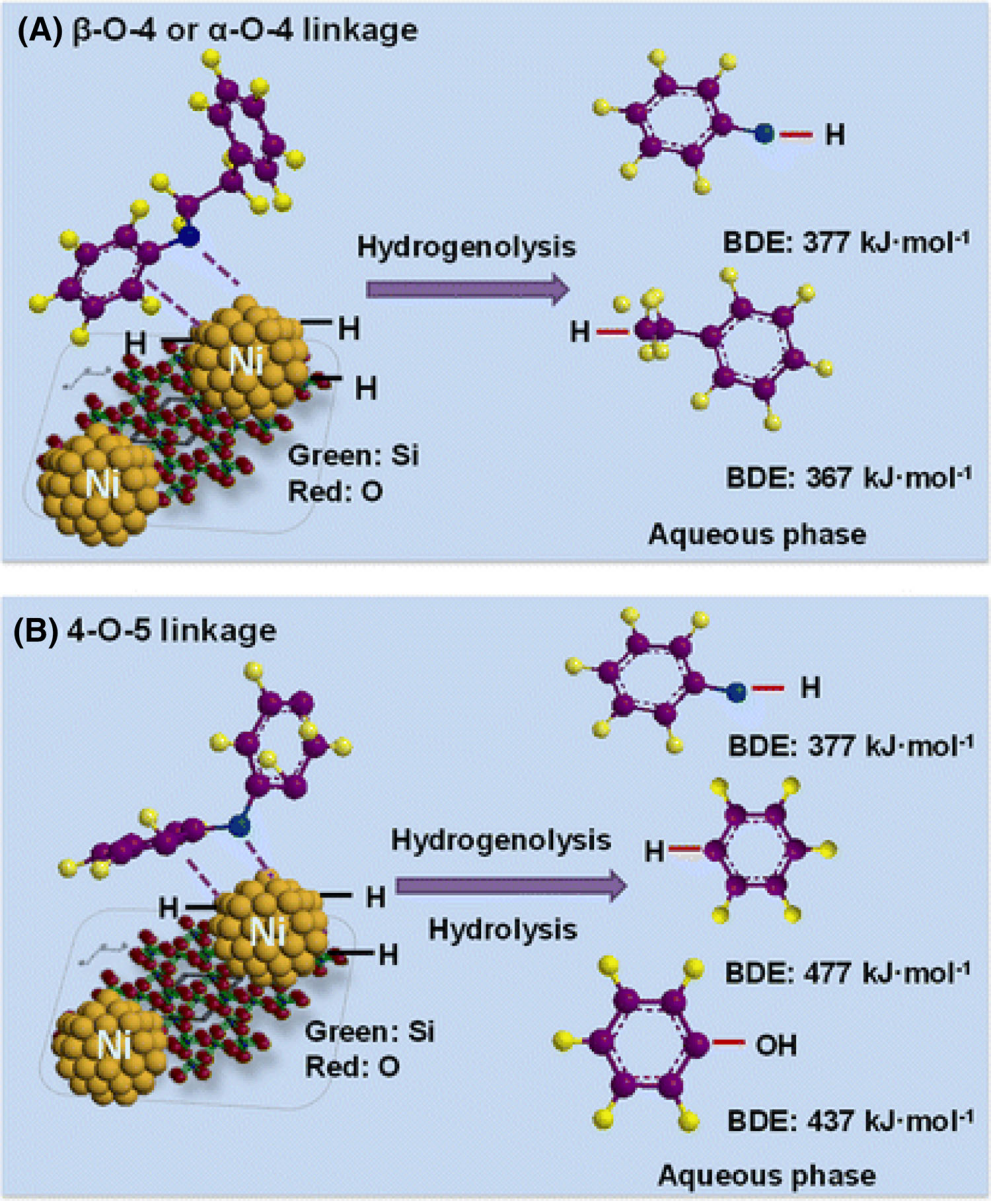
Proposed mechanism for C–O cleavage in lignin model compounds over Ni/SiO_2_ catalysts. Reproduced with permission from Ref. [26]. Copyright © 2012 American Chemical Society

He et al. [26] studied the cleavage of aromatic ether linkages by a nickel catalyst in model lignin compounds, such as benzyl phenyl ether, 2-phenylethyl phenyl ether, and diphenyl ether, which contain β-O-4, α-O-4, and 4-O-5 bonds, respectively. These linkages could be selectively cleaved over Ni/SiO_2_ to yield monomeric aromatic compounds, such as cycloalkanes and cyclohexanol, under moderate conditions (120 °C and 6 bar H_2_ in aqueous medium). Two different mechanisms were proposed depending on the bond type, as illustrated in Fig. 2. Aromatic ethers containing β-O-4, α-O-4 bonds underwent hydrogenolysis into monomeric units, while 4-O-5 bonds were broken by competing hydrolysis and hydrogenolysis pathways (see Fig. 3).

**Fig. 4:**
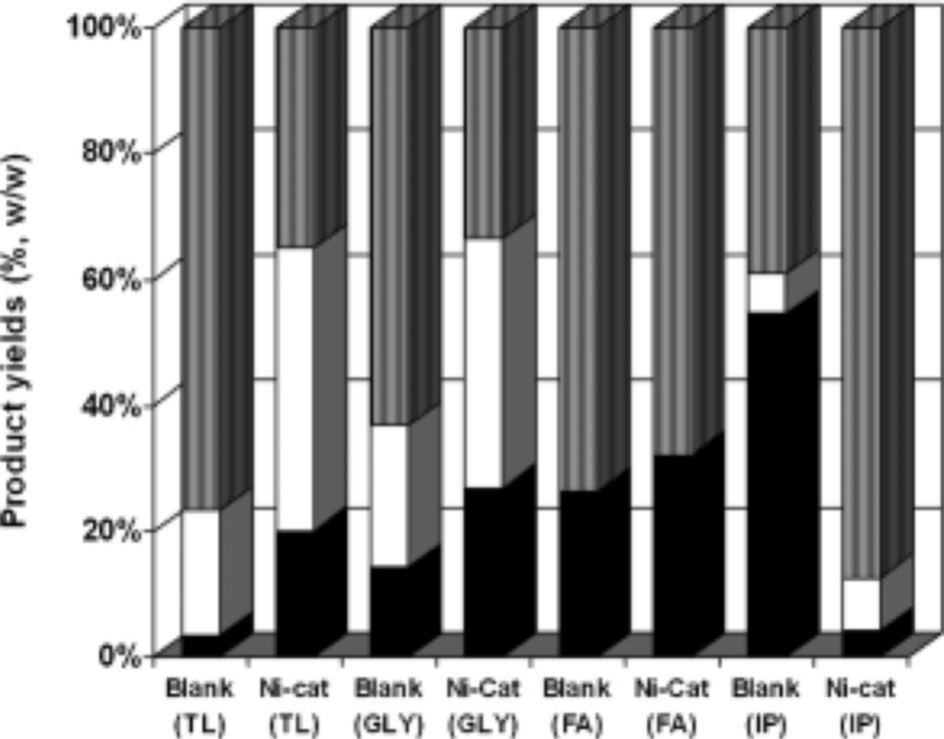
Product yields for the catalytic hydrogenolysis of lignin over Ni catalysts in different hydrogen-donor solvents (*TL* tetralin, *GLY* glycerol, *FA* formic acid, and *IP* isopropanol; *black square* bio-oil, *white square* biochar, *grey square* residual lignin). Reproduced from Ref. [30] with permission of John Wiley & Sons 2013

Supercritical solvents appear a favourable media for lignin depolymerization due to their advantageous properties in terms of hydrogen-donor capacity, rapid heat transfer, and high lignin solubility, with the product distribution strongly dependent on the catalyst/solvent combination. The formation of char by-products through repolymerization is reduced by the use of noble metal and nickel catalysts [27], in the presence of supercritical methanol, ethanol, and propanol, employing molecular H_2_ at 350 °C. The highest lignin oil yield was obtained for a Pt/C catalyst and ethanol solvent, and comprised monomeric phenols, including 4-ethylphenol, guaiacol, 4-ethylguaiacol, and syringol, along with heavier phenolic compounds. The combination of hydrogen in the gas phase and H-donor solvents proved beneficial, favouring higher yields of monomeric phenols due to the quenching of free radicals and concomitant hampering of their recombination into oligomeric phenols [9]. Rinaldi et al. systematically investigated the effect of solvent choice on lignin conversion [14], finding that Lewis basic solvents, such as methanol and 1,4-dioxane, suppressed hydrogenation of aromatic products. In addition, lignin depolymerization was possible in solvents, in which it was insoluble due to the solubility of aromatic fragments formed in situ by lignin thermolysis around 250 °C, which subsequently underwent hydrogenolysis. However, lignin functionalities, such as alcohols lowered catalytic activity due to their preferential binding at catalyst active sites. The use of H-donor solvents without molecular hydrogen for the hydrogenolysis of lignin has also been explored by Toledano and co-workers over bifunctional Ru, Pt, Pd, and Ni catalysts on a mesoporous aluminosilicate Al-SBA-15 support [28], prepared by ball-milling to maximize the accessibility of active sites which are predominantly located on the external surface [29]. Microwave irradiation was also utilised to lower the reaction temperature for catalytic lignin hydrogenolysis of lignin to 150 °C. Nickel containing catalysts proved the most active, offering the highest conversion towards bio-oil (which mainly comprised phenolic monomers), as compared with noble metal catalysts, with the highest yield achieved for a 10 wt% Ni catalyst and tetralin H-donor solvent. The major monomeric phenols afforded by Ni catalysts under microwave irradiation were syringol, syringaldehyde, vanillin, and (des)aspidinol, with syringyl derivatives dominant. The higher activity of Ni catalyst was attributed to their larger particle size of 35–40 nm than of corresponding noble metals (around 5–6 nm), which provided for increased interactions between the parent lignin and metal active sites; Ni catalysts also suppressed repolymerization. The performance of the optimum 10 wt% Ni/Al-SBA-15 catalyst was then subject to a systematic study on the impact of H-donor solvent selection, including formic acid, tetralin, isopropanol, and lignin. Figure 4 shows the resulting distribution of products for each solvent. The yield of the desired bio-oil was high for formic acid, with minimal char formation and syringol as the major product (which was not observed when using isopropanol), the different product selectivities were attributed to differing rates of in situ hydrogen formation.

The combination of Raney Ni with an acidic zeolite also exhibited a synergetic effect in the depolymerization of a cellulolytic enzyme lignin using a methanol/water solvent mixture at 250 °C [31]. The monophenol yield increased from 12.9 wt% when the reaction was catalyzed by Raney Ni alone, to 21–27.9 wt% when used in conjunction with an acid catalyst. Lignin depolymerization into aromatic monomers over solid acid catalysts, such as zeolites, clays, and sulphated zirconia, was evaluated by Deepa and Dhepe [32]. It was proposed that the improved yield from combining Raney Ni with a zeolite arose from the latter hindering the reaction between the parent lignin and other more reactive lignin fragments that could together to produce high molecular weight oligomers. Bimetallic NiM (M = Ru, Rh, Pd, Au, Mo) catalysts have also been studied for lignin hydrogenolysis [33, 34], [35] [36], wherein NaOH addition proved beneficial, promoting the formation of aromatic compounds and supressing arene hydrogenation. Konnerth et al. [37] also observed increased Organosolv lignin depolymerization via NaOH addition to the aqueous reaction mixture containing an Ni_7_Au_3_ nanoparticle catalyst. It was speculated that the basic conditions favoured the formation of phenolate products, whose superior solvation inhibited their subsequent absorption and hydrogenation.

Molybdenum catalysts have also been studied for lignin depolymerization over different supports, including alumina and carbon [38, 39]. Ma et al. reported lignin depolymerization in supercritical ethanol over Mo/Al_2_O_3_ and the optimum reduction temperature evaluated and identified as 750 °C. Lignin was converted into a complex mixture of low molecular weight compounds, such as aliphatic alcohols, C_8_–C_10_ esters, monophenols, benzyl alcohols, and arenes [39]. The same authors reported that nanostructured Mo_x_C achieved complete catalytic ethanolysis of Kraft lignin in supercritical ethanol under N_2_ at 280 °C [38]. Hydrogen pressure exerted a moderate influence on the yield of liquid products, increasing alcohol production at the expense of esters. The principal products were aliphatics (C_6_ alcohols and C_8_–C_10_ esters), and aromatics, including phenols, phenyl alcohols, and C_8_–C_10_ arenes, with negligible dimers, oligomers or char observed. Ethanol provided superior a liquid product yield to water, methanol, or isopropanol, with a complex interplay between the solvent, lignin source and reaction conditions. The small molecules produced through this route are amenable to the direct introduction to a petroleum refinery without further upgrading.

**Fig. 5:**
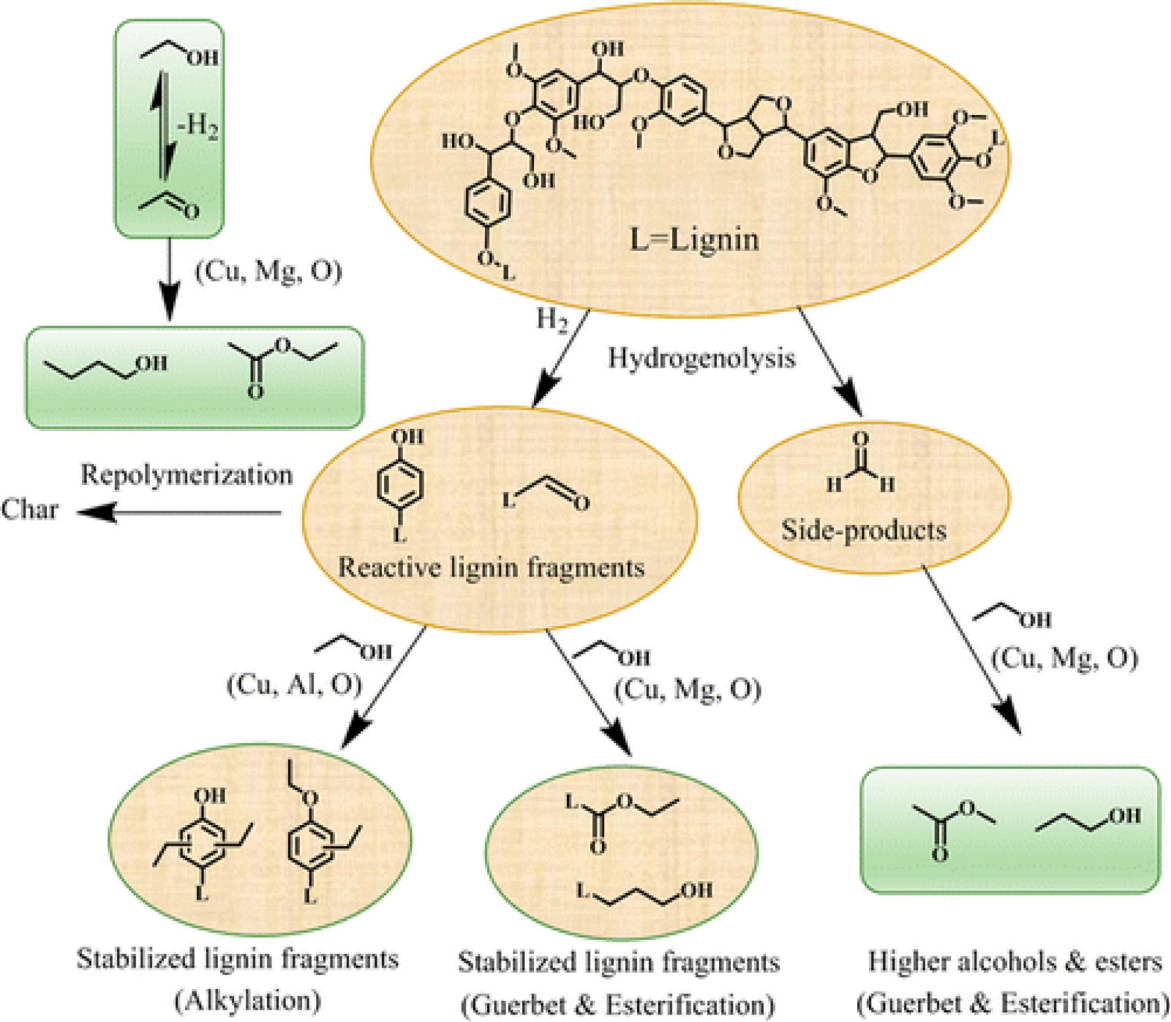
Proposed reaction scheme for lignin depolymerization in ethanol over a Cu_*x*_MgAl_*y*_ catalyst. Reproduced from Ref. [40] with permission. Copyright © 2015 American Chemical Society

The role of each component within a Cu–Al–Mg mixed oxide catalyst for lignin depolymerization was investigated by Jensen et al. [40]. Here, ethanol participated as both solvent and an alkylation agent, reacting with higher alcohols, esters, aldehydes, ethers, and other small hydrocarbons, as shown in Fig. 5. The conversion of lignin into monomers is increased by the combination of Cu with and Lewis acid sites, when is supported over alumina, that increase the amount of monomers and quench the repolymerization. The combination of Cu with basic sites prompts ethanol dehydrogenation producing in the H_2_ necessary for hydrogenolysis and hydrodeoxygenation. In addition, the formation of char is reduced through Guebert reaction and esterification reactions, as it can be observed in scheme. The optimum composition found for the catalyst was a 20 wt% of Cu originated from a hydrotalcite with (Cu + Mg)/Al ratio of 4 gave the highest monomers yield and the char formation by repolymerization degree was lower.

Char formation during lignin depolymerization is highly undesirable. Cu-doped porous catalysts in supercritical methanol reportedly suppress chars, with hydrogen obtained by in situ solvent reforming [41]. A similar approach was adopted by Anastas et al. [42], employing porous metal oxides containing Cu^2+^, Mg^2+^, and Al^3+^, achieving almost complete lignin conversion to catechols under mild conditions of 140–220 °C. The role of Cu as a char inhibitor was attributed to enhanced hydrogenolysis, dehydration, and hydrogenation steps after cleavage of β-O-4 linkers, eliminating product aromaticity [41, 42].

Klamrassamee et al. incorporated ZrO_2_ in MCM-41 and SBA-15 via wet impregnation as supports for the depolymerization of Organosolv lignin using methanol or water as solvents [43]. Zirconia was selected due to it hydrothermal stability and acidity, with the supports chosen for their mesoporous textural properties. Organosolv lignin was depolymerized at 250 °C under an atmospheric pressure or hydrogen, with MCM-41 and SBA-15 supported zirconia offering superior conversion to other ZrO_2_ analogues, attributed to the higher acidity of the silica supports.

### Pyrolysis

Lignin transformation into monomeric units, such as aromatic hydrocarbons, can also be achieved via catalytic pyrolysis, wherein the use of a catalyst affords more heavily deoxygenated and less acidic bio-oils than thermal pyrolysis, thus improving their stability and heating value. In catalytic biomass pyrolysis, the vapours generated in the pyrolysis process are passed through a catalyst bed with a view to promoting deoxygenation through, e.g., decarbonylation, decarboxylation, or dehydration reactions. Lignin requires higher temperatures and long residence times than for catalytic pyrolysis of cellulosic materials [44]. Since lignin is the most thermally stable of the lignocellulosic biomass components, reaction temperatures typically exceed those of 500–550 °C required for cellulose and hemicellulose [45]. The major products of lignin pyrolysis are phenolic compounds, such as phenol, guaiacol, and syringol [46–48].

**Fig. 6:**
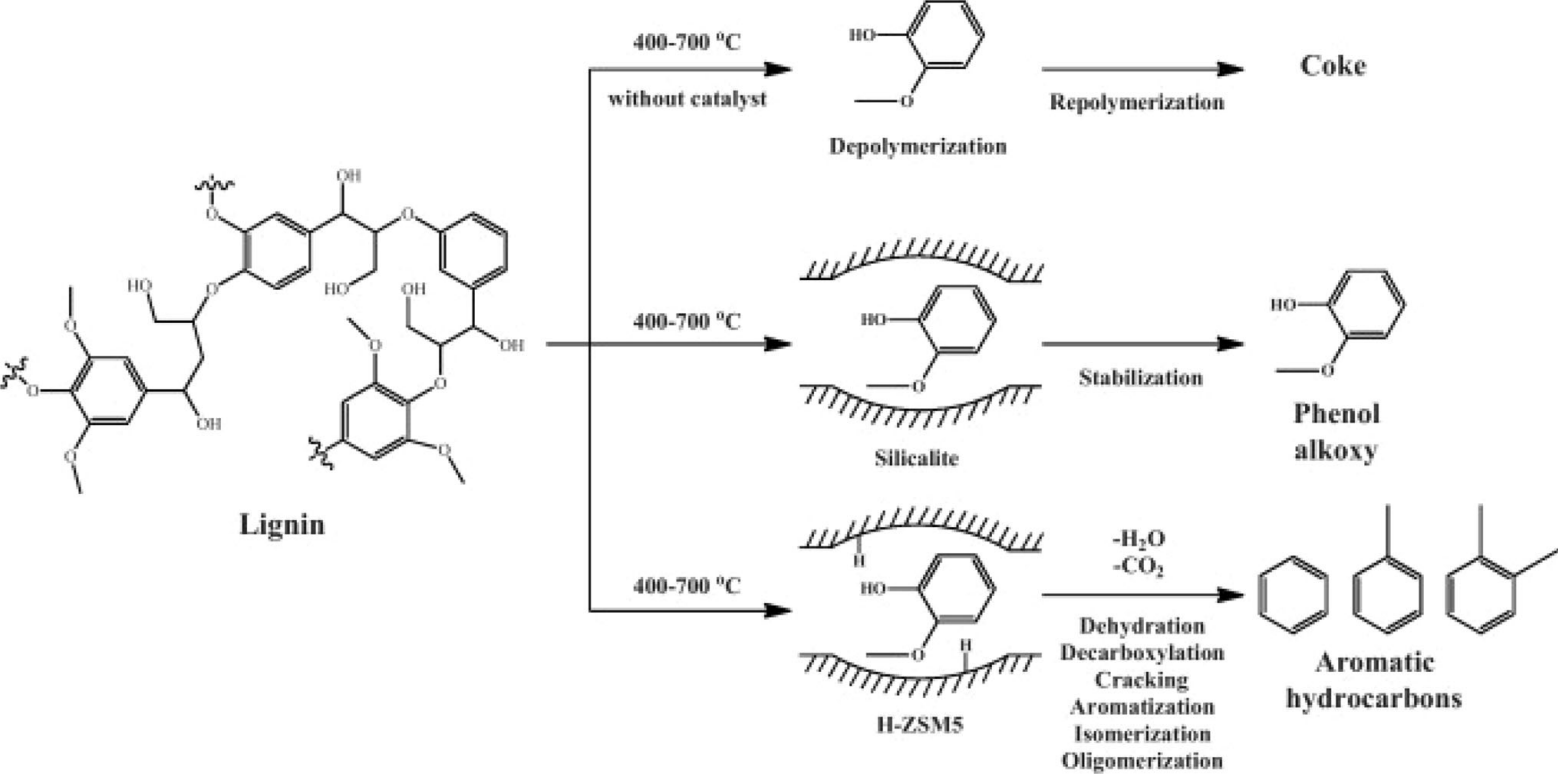
Competing reaction pathways in thermal and catalytic fast pyrolysis of lignin over zeolites. Reproduced from Ref. [52] with permission from Elsevier

The composition of bio-oils from lignin pyrolysis is influenced by the choice of catalyst and its physicochemical properties, notably the pore dimensions and nature of active sites. In the presence of zeolites, a low Si/Al ratio and concomitant high density of acid sites yield high quantities of aromatic compounds. Larger pore sizes are able to accommodate bulkier molecules and deliver stable compounds, with Agrawal et al. [49] studying the influence of such parameters in the fast pyrolysis of alkaline lignin, achieving the highest yield of phenol alkoxyls for a ZSM-5 zeolite, while a USY zeolite favoured aromatic hydrocarbons possessing the highest number of acid sites of all zeolites examined (silicalite, Beta zeolite, and ZSM-5 zeolites with Si:Al ratios from 15 to 210). Brönsted acid sites within ZSM-5 zeolites are held responsible for cracking and reforming reactions [50], explaining the propensity for materials with low Si:Al ratios (high Brönsted acid densities) to favour aromatic products [45, 49]. The impact of zeolite pore size and coke formation has also been explored over ZSM-5, mordenite, Beta, and Y zeolites, which possess different pore architectures, but similar pore dimensions of between 5.6 and 7.6 Å [49, 51]. Coking during lignin pyrolysis is considered a consequence of repolymerization reactions within zeolite pores. Zeolites must, thus, offer high activity towards lignin depolymerization into the desired compounds, while stabilising the products towards undesired coke formation (see Fig. 6).

A range of metal oxides (Co_3_O_4_, MoO_3_, NiO, Fe_2_O_3_, MnO_3_, and CuO), mixed metal oxides, such as CoO/MoO_3_, and supported metal oxides have also been explored for lignin pyrolysis [48, 53]. In general, the liquid bio-oil yield was higher for all catalysts than for thermal pyrolysis, with phenol alkoxy compounds as the primary products over the metal oxides. Nickel and manganese oxide offered a similar product distribution to thermal pyrolysis, with 2-methoxy phenol favoured by NiO and CuO, and MoO_3_ favouring vanillin instead of phenols and 2-methoxy phenols. The yield of aromatic hydrocarbons was increased by supporting nickel or cobalt oxides over ZSM-5 [53], which promoted direct deoxygenation of the aromatic lignin backbone compared to the unsupported metal oxide counterparts.

### Base-catalyzed depolymerization

Lignin depolymerization over base catalysts is an excellent route to monomeric phenols. Aryl–alkyl (β-O-4) ether bonds in lignin are easier to cleave than C–C bonds in base-catalyzed lignin depolymerisation [54], although mineral bases typically require harsh reaction temperatures of around 340 °C [55–57], which can generate significant gaseous products via side reactions and consequent lower monomeric hydrocarbon yields [58]. In base-catalyzed depolymerization, the choice of lignin influences the yield of bio-oil, but not the resulting composition [59]; however, the use of mineral bases may necessitate neutralisation of the subsequent bio-oil, in addition to reactor corrosion. Thring et al. studied the effect of time and temperature on the depolymerization of Alcell lignin catalyzed by NaOH [57] obtaining guaiacol, syringol, and under certain conditions catechol and its derivatives [57, 60, 61]. Base-catalyzed depolymerization has proven an effective protocol in polar organic solvents, such as ethanol and methanol, suppressing char formation, and hence enhancing monomer and dimer production relative to solid acid catalysts. Toledano et al. [56, 62] reported that base catalysts prevent the repolymerization of monomeric products from lignin, offering improved bio-oil yields. Base strength plays an important role on the depolymerization mechanism, with hydroxides promoting phenol, cresol, guaiacol, catechol, and 4-methylcatecol production, while neither catechol or cresols were observed when using potassium carbonate [56]. The use of capping agents, such as boric acid, to protect hydroxyl groups in phenolic compounds has also been explored in efforts to minimize repolymerization, and results in lower molecular weight products [60]. In alkaline media, boric acid reacts with phenolic monomers to produce their respective esters (NaB(OR)_4_), which hinders side reactions of phenol products and, in turn, hinders char formation. The formation of borated esters during base-catalyzed depolymerization also offers higher yields as compared with boric acid protection alone under analogous conditions [62]. The use of phenol was also explored as a capping agent to stabilise monomeric products of lignin depolymerization, with catechol and ferulic acid formed, whereas capping by boric acid yielded significant dimers. Toledano et al. [62] suggested that the different behaviour of these capping agents were due to boric acid trapping intermediates which lowered the yield of monomeric aromatics, whereas phenol capping reduced oligomerization, but not demethoxylation and dealkylation pathways.

MgO is a widely used solid base catalyst for biodiesel synthesis [63], and has also been employed for lignin depolymerisation in a range of solvents, including water, methanol, ethanol, and tetrahydrofuran (THF) [64]. Maximal lignin conversion was achieved for THF due to its superior solubilisation of lignin; however MgO also catalyzed repolymerization of phenol oligomers. Other solid bases, such as RbCO_3_ CsCO_3_, have also shown promise for β-O-4 cleavage of ether bonds in lignin model compounds, but are able to suppress char formation and increase bio-oil yield [65, 66].

Considering that lignin depolymerization can be performed successfully with homogeneous base catalysts, such as KOH and NaOH, with conversions reaching 95 % [55], [60], and that heterogeneous Ni catalysts are able to cleave C–O bonds [67], Sturgeon et al. proposed a combination of these active components [19]. A 5  wt% Ni supported on a MgAl hydrotalcite (a solid base catalyst employed widely for lipid transesterification to biodiesel [68–70]) was shown to depolymerise a lignin model compound, 2-phenoxy-1-phenethanol, and ball-milled lignin extracted from corn stover, into low molecular weight alkyl aromatics; interestingly, nickel nanoparticles present on the (likely external) hydrotalcite surface required no activation with H_2_ prior to their catalytic application, suggesting that the active species was a mixed valence nickel oxide. Catalytic properties of nickel hydrotalcites are strongly influenced by the nature of interlayer anion. The presence of NO_3_^−^ anions, or other basic anions, such as OH^−^ and CO_3_^2−^, in the interlayers conferred up to a three-fold activity enhancement in the depolymerisation of the model lignin compound 2-phenoxy-1-phenethanol [71], wherein acetophenone (and phenol) were the principal products. Although the origin of this base promotion remains unclear, the depolymerisation mechanism may involve direct nitration of the phenolic. The same nitrate intercalated Ni hydrotalcite also proved effective for the depolymerization of enzymatically hydrolyzed lignin from corn stover, resulting in a large proportion of low molecular weight products, predominantly phenol, guaiacol, and syringol monomers, when performed in water, as compared with 4-vinylphenol when conducted in 3-methyl-3-pentanol at temperatures <200 °C.

### Oxidative transformations

Oxidative depolymerization is often considered undesirable due to the propensity for generating free radicals which may promote repolymerization of the desired mono/dimeric products and consequent char production. However, oxidative protocols can be advantageous, since they permit retention of both aromatic and acyclic organic frameworks without C–C bond scission. Oxidative degradation is the route adopted for lignin decomposition in the natural world [72], and offers a hydrogen-free pathway to vanillin [11, 73]. A range of organometallics (notably methyl trioxo rhenium), polyoxometalates, and vanadium catalysts are reported for the oxidative depolymerization of lignin. Vanadium–oxo complexes have also been demonstrated as efficient catalysts for C–O bond cleavage in lignin model compounds [74]. Silk et al. showed that the choice of vanadium complex strongly influenced selectivity, directing either the cleavage of C–C bonds between an aryl ring and the contiguous group, or C–O linkages scission [75]. Polyoxometalates are robust redox catalysts, and effective for oxidative degradation; when performed in the presence of molecular oxygen, a two-step process occurs, in which the polyoxoanion is first reduced and, subsequently, reoxidised without any net structural change [76]. Polyoxometalates applied for the decomposition of lignin residues under molecular oxygen often yields CO_2_ and H_2_O by-products [77]. Dimethyl succinate and aromatics have also been produced from pyrolytic lignin using a vanadium/molybdenum polyoxometalate in a methanol/water solvent mixture under oxygen [72]. Bio-oil production increased with the amount of methanol (or ethanol) via methanol oxidation to CCH_3_ and CH_3_OC radicals which promoted lignin depolymerization. However, the addition of methanol (or ethanol) under alkaline conditions did not favour monomeric products [78]. The major products using a H_5_PMo_10_V_2_O_40_ catalyst were organic acids and their esters, such as dimethyl fumarate and dimethyl succinate, formed by the breakdown of aromatics [79].

Salen complexes, particularly of cobalt, have also been tested for oxidative lignin transformation by H_2_O_2_ or molecular oxygen. Copper complexes, such as Cu-bipy and Cu-phen, can also catalyse the oxidative transformation of lignin model compounds, such as veratryl alcohol and 2,2-biphenol. Oxidation activity was a function the of steric and electronic ligand effects [75]. Hydrogen peroxide is widely used in organic transformations and, generally, considered as a green oxidant with water as the sole by-product [80]. The in situ production of reactive oxygen species from peroxy species by reaction with Cu or Fe is termed the Fenton reaction [81], and been utilised for the degradation of veratryl alcohol [82].

A chemoselective method for the aerobic oxidation of secondary benzylic alcohols, in the presence of unprotected primary alcohols (abundant in lignin), has also been developed by combining 4-acetamido-TEMPO (as a radical oxidant) together with HNO_3_ and HCl [83, 84]. This approach has been applied to wood pulp delignification, in which oxidation of the C_α_ alcohol to a ketone favours the breakdown of β-O-4 linkages. Wang et al. [85] also reported a bifunctional Pd/CeO_2_ catalyst able to efficiently cleave β-O-4 bonds in the presence of C_α_-hydroxyl group, in which synergy between palladium activation of the oxidant and the redox properties of ceria facilitates β-O-4 cleavage; Pd is widely used in heterogeneous catalysis for aerobic alcohol oxidation [86–90]. This bifunctional catalyst was able to transform a range of Organosolv lignin in ethanol under oxygen to vanillin, guaiacol, and 4-hydroxybenzaldehyde.

Oxidative cleavage of a dilignol model compound, erythro-1-(3,4-dimethoxyphenyl)-2-(2-methoxyphenoxy)-l,3-propanediol, was investigated employing transition metal containing hydrotalcite, and V(acac)_3_ and Cu(NO_3_)_2_.3H_2_O mixtures, with molecular oxygen as the oxidant [91]. Oxidative depolymerization of the dilignol model was influenced by the solvent selection, with negligible conversion observed in toluene, but good activity obtained in pyridine resulting in veratric acid as the major product. The same catalysts were subsequently applied to both Organosolv and Kraft lignin; however, their performance could not be readily extrapolated from dilignol model due to the complexity of practical lignin sources and presence of zeolite contaminants; harsher reaction conditions (extended reaction duration and higher oxygen pressure) were required to depolymerize Organosolv lignin, even at lower concentrations than used for the dilignol model, although both Cu-V hydrotalcite and V(acac)_3_/Cu(NO_3_)_2_·3H_2_O successfully cleaved β-O-4 linkages and resinol components yielding dimeric and trimeric products.

### Use of ionic liquids

Ionic liquids (ILs) have attracted a great deal of interest in recent years as green solvents suitable for the solubilisation of lignocellulosic biomass, that are insoluble in other media [92, 93]. After dissolution in an IL, biomass is amenable to acid hydrolysis [94], acylation [95], and acetylation [96]. Selection of the appropriate IL is key to control the product selectivity in oxidative depolymerization [97]. Lignin depolymerization has proven possible under mild conditions of only 110–150 °C through the use of an acidic IL, 1-H-3-methylimidazolium, via hydrolysis of ether bonds [98]. The most widely used ILs are 1-ethyl-3-methylimidazolium (EMIM) and 1-butyl-3- methylimidazolium (BMIM) with anionic counterions, such as trifluoromethanesulfonate [CF_3_SO] [99]. Bosmann et al. [97] evaluated the choice of IL in the oxidative depolymerization of lignin catalyzed by iron, copper, and manganese salts. Highest conversion was found for Mn(NO_3_)_2_ in [EMIM][CF_3_SO] after 24-h reaction at 100 °C and 8.5 bar air, yielding 2,6-dimethoxy-1,4-benzoquinone (DMBQ), and either syringaldehyde or vanillic acid [97]. Cobalt salts favoured the oxidation of benzyl and other alcohol functionalities within lignin after its complete dissolution in an IL, such as 1-ethyl-3-methylimidazolium diethylphosphate [EMIM][DEP], without modifying phenolic functionalities or 5-5′ β-O-4 phenilcoumaran bonds, explaining the absence of monomeric products [100]. [EMIM][DEP], thus provide an efficient medium for lignin oxidative degradation.

### Enzymatic lignin depolymerization

**Fig. 7:**
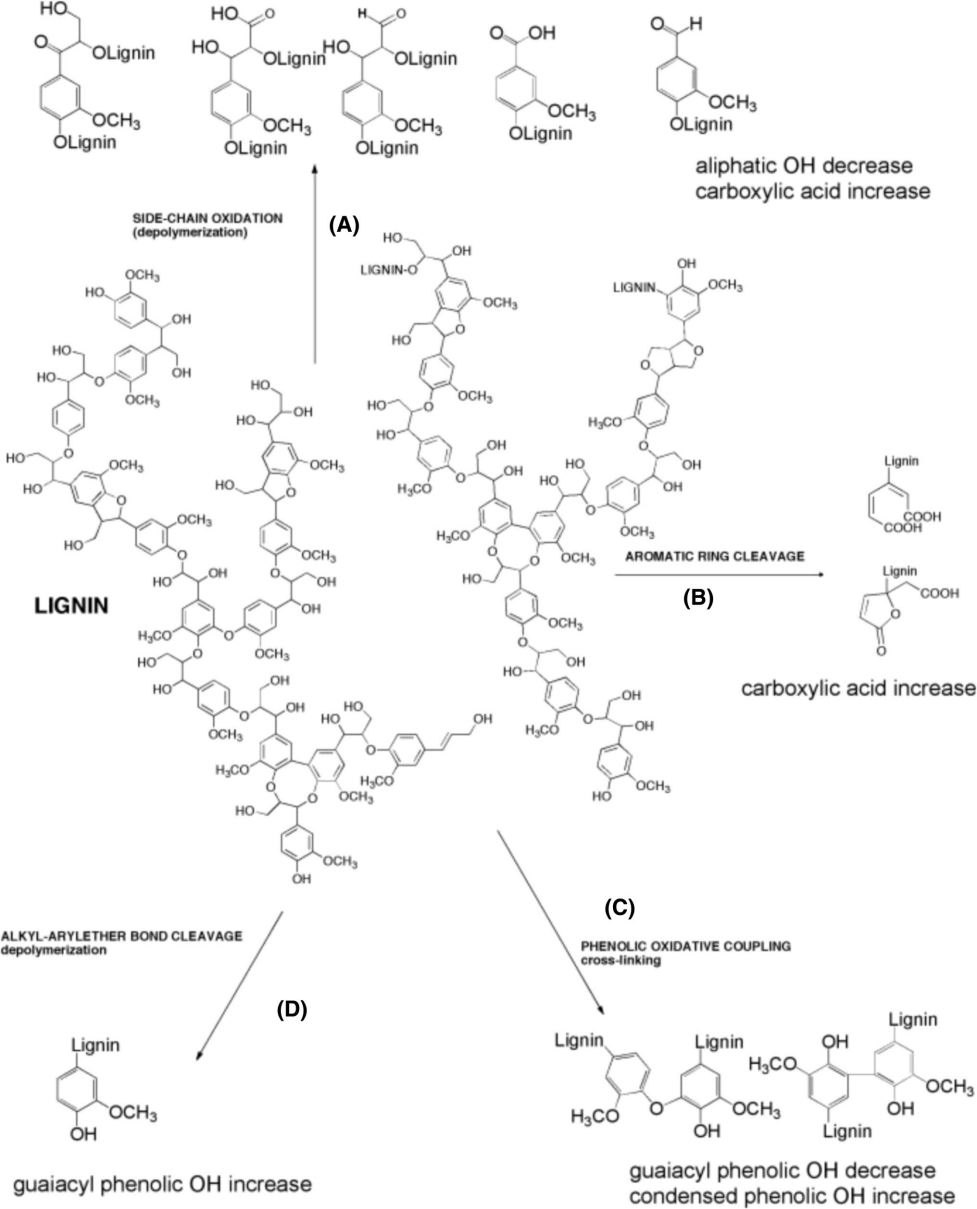
Possible routes for the enzymatic oxidative depolymerisation of lignin. Reproduced from Ref. [105] with permission from Elsevier

Oxidative lignin depolymerization has also been catalyzed by enzymes, such as peroxidases [101, 102] and laccase, which are lignolytic enzymes [15]. Laccase and peroxidase are useful enzymes due to their low substrate specificity (i.e., broad chemical applicability) and wide range of pH stability [103], contrasting the general perception that enzymes have limited applicability in chemical synthesis due to their high specificity and narrow pH dependence [104]. Oxidative lignin degradation employing the peroxidase *Pleurotous ostreaus* was shown at 30 °C, under molecular O_2_ in a pH 4 buffer solution, yielding 2,6-dimethoxy-1,4-benzoquinone, benzoic acid, butyl phthalate, and bis(2-ethylhexyl) phthalate. The decomposition mechanism was proposed to initiate via a phenoxy radical, formed by the oxidation of a free phenolic group, which promoted C_α_–C_β_, cleavage, the oxidation of C_α_ carbons, and β-O-4 linker scission [102]. Enzyme immobilization has also been attempted to improve product separation and catalyst recovery and reuse from water. Horseradish peroxidase (HRP) have been chemically immobilised on alumina using a layer-by-layer methodology and applied to the oxidative functionalization of Kraft lignin residue, and exhibited superior depolymerization to the free enzyme [105], attaining 90 % lignin conversion versus 50 % for the native enzyme. This enhancement was attributed to the enzyme stabilization provided by the electrolyte coating in the support employed during the immobilization process. In this case, depolymerization was believed to compete with oxidative coupling reactions which consumed residual insoluble lignin resulting in the formation of higher molecular weight polymers. The amount of carboxylate groups increased without any change in the aliphatic hydroxyl groups, indicating that side chains were not oxidised. Figure 7 shows a tentative scheme for enzymatic lignin oxidation proposed by Perazzini et al. [105]. A multi-enzyme heterogeneous catalyst was created through the immobilization of horseradish and laccase enzymes over alumina, and evidenced a synergy between the two enzymes [103]. The high concentration of aliphatic and phenolic functions present in lignin suggested a hydrolytic process as the responsible for its depolymerization.

## Conclusions

Lignin represents an important renewable resource for the production of chemicals and bio-fuels, and hence, its valorisation will play an increasingly important role in the development of sustainable biorefineries. However, the heterogeneous nature of lignin presents significant obstacles for the establishment of a standard methodology for its depolymerization. The development of novel catalytic routes to lignin depolymerisation will facilitate more atom-efficient and energy-efficient processes, and safer approaches, such as the use of hydrogen-donor solvents instead of high-pressure molecular hydrogen. Heterogeneous catalysts will also afford facile catalyst separation/reuse and product purification. Alternative solvents, such as ILs, are promising for low-temperature lignin decomposition in conjunction with the conventional mineral acids or tailored homogeneous/heterogeneous catalysts, although the high price and potential corrosiveness and toxicity of some ILs may limit their industrial scale application. The aromatic fraction produced through catalytic lignin depolymerization still exhibits a comparatively low heating value and high acid content, reflecting the high proportion of oxygenated compounds present in the bio-oil, and concomitant poor miscibility with aliphatic hydrocarbons, in addition to a broad boiling point range. Bio-oil upgrading technologies will, thus, be essential for either fuel or chemical applications; of these, hydrodeoxygenation (HDO) will likely prove most successful for biofuel, affording the complete hydrogenation of oxygenated depolymerisation products to benzene and cyclohexane.
